# High prevalence of epilepsy in onchocerciasis endemic health areas in Democratic Republic of the Congo

**DOI:** 10.1186/s40249-018-0452-1

**Published:** 2018-08-01

**Authors:** Evy Lenaerts, Michel Mandro, Deby Mukendi, Patrick Suykerbuyk, Housseini Dolo, Deogratias Wonya’Rossi, Françoise Ngave, Chellafe Ensoy-Musoro, Anne Laudisoit, An Hotterbeekx, Robert Colebunders

**Affiliations:** 1 Global Health Institute, University of Antwerp, Antwerp, Belgium; 2 Provincial Health Division of Ituri, Ministry of Health, Bunia, Democratic Republic of Congo; 3 Centre Neuro Psycho Pathologique, Université de Kinshasa (CNPP-UNIKIN), Kinshasa, Democratic Republic of Congo; 4 International Center of Excellence in Research, Faculty of Medicine and Odontostomatology, Bamako, Mali; 5 Neglected Tropical Diseases Control program, Ministry of Health, Bunia, Democratic Republic of the Congo; 6 Centre de Recherche en Maladies Tropicales, Hôpital Général de Référence de Rethy, Rethy, Democratic Republic of Congo; 7 Interuniversity Institute for Biostatistics and statistical Bioinformatics, University of Hasselt, Hasselt, Belgium; 8 EcoHealth Alliance, New York, USA; 9 Global Health Institute, Faculty of Medicine and Health Sciences, Gouverneur Kinsbergen Centrum, Doornstraat 331, 2610 Wilrijk, Belgium

**Keywords:** Onchocerciasis; epilepsy, Prevalence, Democratic Republic of Congo

## Abstract

**Background:**

A high prevalence of epilepsy has been observed in many onchocerciasis endemic regions. This study is to estimate the prevalence of active epilepsy and exposure to *Onchocerca volvulus* infection in a rural population in Ituri province, Democratic Republic of Congo.

**Methods:**

In August 2016, a community-based cross-sectional study was conducted in an onchocerciasis endemic area in the rural health zone of Logo, Ituri Province. Households within two neighbouring health areas were randomly sampled. To identify persons with epilepsy, a three-stage approach was used. In the first stage, all individuals of the selected households were screened for epilepsy by non-medical field workers using a validated 5-item questionnaire. In the second and third stage, suspected cases of epilepsy were examined by non-specialist medical doctors, and by a neurologist, respectively. A case of epilepsy was defined according to the 2014 International League Against Epilepsy (ILAE) guidelines. Exposure to *O. volvulus* was assessed by testing for IgG4 antibodies to an *O. volvulus* antigen (OV16 Rapid Test,) in individuals aged 3 years and older.

**Results:**

Out of 1389 participants included in the survey, 64 were considered to have active epilepsy (prevalence 4.6%) (95% confidence interval [*CI*]: 3.6–5.8). The highest age-specific epilepsy prevalence estimate was observed in those aged 20 to 29 years (8.2%). Median age of epilepsy onset was 10 years, with a peak incidence of epilepsy in the 10 to 15 year-old age group. OV16 test results were available for 912 participants, of whom 30.5% (95% *CI*, 27.6–33.6) tested positive. The prevalence of OV16 positivity in a village ranged from 8.6 to 68.0%. After adjusting for age, gender and ivermectin use, a significant association between exposure to onchocerciasis and epilepsy was observed (adjusted odds ratio = 3.19, 95% *CI*: 1.63–5.64) (*P* < 0.001).

**Conclusions:**

A high prevalence of epilepsy and a significant association between epilepsy and exposure to *O. volvulus* were observed in the population in Ituri province, Democratic Republic of Congo. There is an urgent need to implement a CDTI programme and to scale up an epilepsy treatment and care programme.

**Electronic supplementary material:**

The online version of this article (10.1186/s40249-018-0452-1) contains supplementary material, which is available to authorized users.

## Multilingual abstracts

Please see Additional file 1 for translations of the abstract into the five official working language of the United Nations.

## Background

In 2016, an estimated 14.6 million people were infected with onchocerciasis worldwide, with over 99% of cases occurring in sub-Saharan Africa [1]. This also includes the Democratic Republic of the Congo (DRC), where onchocerciasis is endemic in all provinces [2].

Onchocerciasis is a parasitic disease caused by infection with the filarial worm *Onchocerca volvulus*, transmitted by blackflies of the genus *Simuliidae* [3]. In infected persons the adult female worms form subcutaneous nodules and release thousands of microfilariae each day, leading to severe itching, disfiguring skin lesions, and visual impairment, including permanent blindness [3].

An increased prevalence of epilepsy has been observed in many onchocerciasis endemic areas [4–8], which has led researchers to hypothesize that epilepsy may be another manifestation of onchocerciasis. This is supported by the observation that nodding syndrome, a neurological syndrome characterized by episodes of atonic seizures, only occurs in regions hyperendemic for onchocerciasis [9].

Community-directed treatment with ivermectin (CDTI) is the cornerstone of efforts to control onchocerciasis [3]. However, despite 16 years of CDTI in several locations in DRC, the therapeutic coverage has not been spatially or yearly consistent [2]. In some areas, there has been no CDTI at all, mainly because onchocerciasis foci can be very localized. Certain villages may not have been included in a CDTI programme if they belonged to a health zone where onchocerciasis was considered hypo-endemic. This is the case in Draju Village, part of Logo health zone (Ituri Province), where Rapid Epidemiological Mapping of Onchocerciasis (REMO) [10] showed that *O. volvulus* nodules were present in 17 of 52 (32.7%) people examined [11]. Although this is clearly above the threshold of 20% required to launch a CDTI programme [10], CDTI has never been implemented in this area.

Moreover, a door-to-door survey in Draju Village in 2015 documented a very high prevalence of epilepsy of 6.2% (95% *CI*, 4.75–7.65) [11]. This prevalence estimate was at least 6 times higher than the prevalence of active convulsive epilepsy documented in 5 African demographic surveillance sites located in non-onchocerciasis endemic areas: 0.78% in Kilifi (Kenya), 0.70% in Agincourt (South Africa), 0.10% in Iganga-Mayuge (Uganda), 0.15% in Ifakara (Tanzania), and 0.10% in Kintampo (Ghana) [12].

In a case-control study performed in Draju Village in 2015, skin snips were positive for *O. volvulus* microfilariae in 55.9% of persons with epilepsy compared to 25.8% in controls, corresponding to an odds ratio (*OR*) of 3.66 (95% *CI*: 1.72–7.78, *P* < 0.001) [13].

Additionally, case-control studies performed in three provinces in the DRC (Bas-Uele, Tshopo and Ituri) suggested that ivermectin might protect against the development of epilepsy in onchocerciasis endemic regions [11, 14]. This finding indirectly supports the hypothesis that *O. volvulus* infection may be an important cause of epilepsy in onchocerciasis endemic areas. However, prospective longitudinal data are required to assess whether the distribution of ivermectin may reduce the incidence of epilepsy in areas endemic for onchocerciasis.

## Methods

### Study objectives

Previous research in Draju Village (Logo health zone) indicated that this area may be an ideal study site for such a future prospective longitudinal study. Therefore, a research team revisited the Logo health zone in August 2016 to determine epilepsy prevalence and onchocerciasis seroprevalence in a larger number of villages in this area. Although not the primary focus of this study, we also examined whether nodding syndrome was part of the clinical spectrum of epilepsy in this area.

### Study setting

**Fig. 1:**
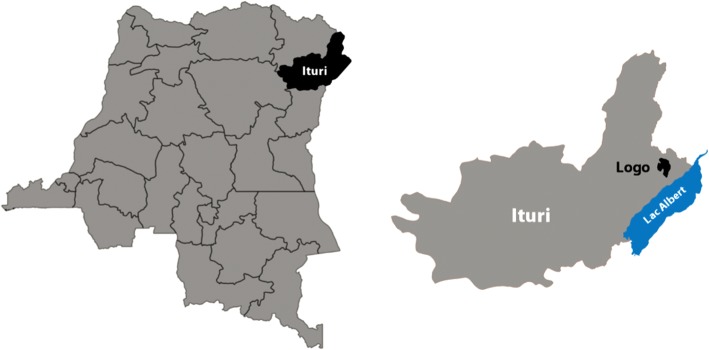
Map of the study area. Location of Ituri Province in the north-east of the Democratic Republic of Congo (left), and location of Logo health zone within Ituri Province, west of Lake Albert (right)

The study was conducted in the health areas of Draju and Kanga, located in the southern part of Logo health zone, Ituri Province, DRC (Fig. 1). The Logo health zone is divided into 26 health areas, dispersed over 284 villages (estimated population 249 220 in 2016). Draju health area includes 13 villages (approximately 7272 inhabitants in 2016); Kanga health area consists of nine villages (approximately 10 109 inhabitants in 2016).

These health areas are on the edge of the Lendu Plateau, with altitudes ranging between 1700 m and 2455 m and located at the northern end of the Albertine Rift, west of Lake Albert in the north-east of the DRC. It borders Uganda in the north, while its eastern part is punctuated by a series of mountains of which Mount Aboro (2455 m) is the highest. Until the 1970s, the Lendu plateau was covered by a dense mountain forest above 1500 m, but is now completely deforested. The plateau landscape is marked by bare soil, agricultural land and grassland with scattered trees; the only forest stretches remaining are riverine galleries extending along the mountain rivers, flowing down from the plateau to the shore of Lake Albert. The Kuda River and its side-streams cross the health areas of Draju and Kanga. They contain several fast-flowing stretches (rapids), which are favourable breeding sites for blackflies, the vectors of *O. volvulus*.

The DRC is one of the poorest countries worldwide with a human development index (HDI) ranked as 178 out of 188 countries listed [15]. Like the rest of the country, the study area is characterized by high poverty rates, high fertility rates, low educational attainment and poor nutrition. The diet of the population lacks variety and is mainly based on the consumption of cassava and plantain bananas. The majority of the population is engaged in agriculture, which is predominantly low-input and subsistence-based. Most people in this area do not have access to proper sanitation facilities, which contributes to a high burden of infectious diseases. Moreover, roads are mostly unpaved, generally in poor condition and largely impassable during the rainy season.

Health care facilities in the Logo health zone lack personnel and equipment, and often run out of critical medicine and supplies. At the time of this study, phenytoin was the only anti-epileptic drug available and only at irregular intervals (Mandro M, personal communication). To date, Logo health zone has never been targeted for CDTI campaigns.

### Participants selection procedure

All permanent households and household members within villages belonging to the health areas of Draju and Kanga were eligible to participate. A household was defined as a person or group of persons ‘living together and eating from the same cooking pot’ [16]. Permanent residents were defined as individuals who had been living in the village for at least 12 months.

Local villagers were recruited to list all households per village and the number of individuals belonging to each household. A random sample of households in each village, proportional to the number of households in each village, was then selected. In these households, all members were eligible to participate. Permission to conduct the study was sought from community leaders prior to approaching households for interviews.

### Epilepsy prevalence and incidence

To identify patients with active epilepsy, a three-stage approach was used. In stage 1, all household members were screened for epilepsy by four teams, each led by an investigator (PS, DH, DR, FN) and accompanied by a local nurse or laboratory technician and a community health worker. In stage 2, a diagnosis of epilepsy was validated by a team of two general practitioners (MM and EL). In stage 3, confirmation of the epilepsy diagnosis was performed by a neurologist (DM). The general practitioners and neurologist were assisted by a translator. The research teams received 2 days of training in the basics of epilepsy, study procedures and the use of data collection tools (tablets).

In stage 1, the screening team visited randomly selected households. The aim and procedures of the study were clarified to the head of the household, or a knowledgeable adult household member if the head of the household was absent. If both were absent, the most adjacent household was chosen.

After informed consent was obtained, information was collected on age, gender, ethnicity and past intake of ivermectin of all household members. All households were located by Geographic Information Systems. To detect persons suspected of having epilepsy, a 5-item previously validated questionnaire [17] was used. This included the following questions: whether a person had ever experienced (1) a loss of consciousness associated with convulsions, loss of urine, drooling and/or tongue biting; (2) sudden and brief loss of contact with their surroundings for a short duration; (3) sudden onset of uncontrollable twitching or abnormal movements of the head, arms, legs of short duration; (4) sudden onset of strange bodily sensations, hallucinations or visual, olfactory or auditory illusions of short duration and (5) whether they had ever been told to have epilepsy.

A positive answer to any one of these questions was used as an indication that a person might have epilepsy; this person or their parents/guardian was/were then asked to be interviewed and examined by a general practitioner (stage 2). During the interview, additional sociodemographic variables were collected, including time of residence in the village, level of education and occupation. To detect possible causes of epilepsy, persons with suspected epilepsy (and/or their mothers) were surveyed about obstetric problems, prematurity, a history of head injury and major infections (measles, malaria, meningitis/encephalitis) and about the presence of epilepsy in other family members. They were also surveyed about the age at onset of first seizures, seizure frequency, precipitating factors, cognitive changes, the use of anti-epileptic drugs and/or traditional therapies. All questionnaires were developed in agreement with local partners and researchers to increase their comprehensibility, completeness, cultural acceptability and feasibility.

During the clinical examination, the following elements were assessed in cases suspected of epilepsy: general condition, weight and height, facial and thoracic abnormalities, absence of external signs of sexual development, signs suggestive of epileptic seizures (burn scars, tongue biting), signs suggestive of onchocerciasis (ocular lesions, visual impairment, pruritic lesions and cutaneous eruptions, leopard and lizard skin, presence and number of subcutaneous nodules) and other neurological conditions (mental retardation, behavioural problems, gait disorders, muscular weakness, paralysis and contractures). If the general practitioner confirmed the diagnosis of suspected epilepsy, the person was referred to the neurologist. This neurologist established a final diagnosis of active epilepsy and determined the type of epilepsy (stage 3).

A case of epilepsy was defined according to the 2014 International League Against Epilepsy (ILAE) guidelines [18]. Individuals were considered to have active epilepsy if: (1) they were on anti-epileptic drugs and had a history of at least one unprovoked seizure, or (2) if patients without anti-epileptic treatment presented at least two unprovoked seizures 24 h apart in the last 5 years. Epilepsy was considered to be resolved for individuals who remained seizure-free for the last 10 years and off anti-epileptic therapy for at least the last 5 years. A diagnosis of probable nodding syndrome was considered if there was a history of head nodding seizures in a previously healthy child who developed nodding episodes between the age of three and 18 years, followed by cognitive impairment and behavioural abnormalities.

Exposure to *O. volvulus* was assessed by a serology-based rapid test (SD Bioline Onchocerciasis IgG4, Standard Diagnostics Inc.) detecting human IgG4 antibodies to the *O. volvulus* antigen OV16 in capillary blood samples in individuals aged 3 years and older.

### Sample size

To calculate an adequate sample size, the following formula was used, assuming simple random sampling: *n* = [(Z_1-α/2_)^2^] × {[p (1 – p)] / d^2^} [19]. For an expected population proportion of epilepsy of 0.03 (based on the results from a previous study in the DRC) [8] and a targeted absolute precision of 0.010 with a 5% error risk, the minimal sample size was estimated at 1118 subjects. A correction of 10% was used to account for potential non-responders, resulting in a sample size of at least 1230 individuals. A mean number of persons per household of six was assumed, resulting in a total selection of approximately 205 households.

### Data collection

Data collection tools were developed in the open source software ‘Open Data Kit’ [20] and EpiCollect+ [21]. Tablets were equipped with a Global Positioning System (GPS) to geolocate the households sampled and to provide accurate estimates of the distribution of cases of epilepsy.

### Data analysis

Continuous variables were analysed using medians and interquartile ranges, whereas categorical variables were analysed using frequencies and percentages. The prevalence of epilepsy was calculated by dividing the number of epilepsy cases confirmed in stage 3 by the total number of individuals screened in stage 1.

To calculate incidence estimates of epilepsy in the study population, cases of epilepsy identified in stage 2 were asked to provide an estimate of the number of months that they had been living with the diagnosis of epilepsy. All subjects with confirmed epilepsy with an onset of seizures within the 12 months prior to the survey, were included into the calculation of epilepsy incidence. Estimates of epilepsy incidence were calculated by dividing the number of incident cases in the previous 12 months by the summed person-years of the population at risk in these 12 months. Person-time at risk was estimated using the following formula: {[(number of people at risk at the beginning of the time interval + number of people at risk at the end of the time interval) / 2] × (number of time units in the time interval)}. Age, gender and area-specific prevalence and incidence rates were calculated.

In order to examine spatial aggregation of epilepsy cases and to test whether any location-specific variables influenced the presence of the disease, a joint point-referenced spatial hierarchical model based on Lindgren et al. [22] was fitted. Presence/absence of epilepsy and OV16 status of the individual were jointly modelled while adjusting for age, gender, and ivermectin use. This model was also used to examine the association between exposure to *O. volvulus* (OV16 seroprevalence) and epilepsy and whether individuals living at higher/lower altitude (per 1000 m) had a higher risk of having epilepsy or being positive for *O. volvulus* infection. Additionally, the effect of the number of neighbouring individuals with epilepsy (within 250 m distance) on the individual risk of having epilepsy or being OV16 positive was investigated. Adjusted odds ratios and their corresponding 95% confidence intervals (*CI*s) were calculated.

To account for the correlation (clustering) of individuals within households, villages and health areas, a household, village and health area random effect was included in the model, as well as a spatially structured random effect to account for the correlation of observations across space. This spatially structured random effect was modelled using a stochastic partial differential equation (SPDE), similar to what is used in geostatistical models to estimate the spatial range (the distance at which spatial correlation is almost negligible and observations can be considered independent). This can be viewed as some sort of spatial aggregation where observations within the spatial range are considered spatially autocorrelated, whereas observations outside the range, are considered independent (correlation < 0.1).

All statistical analyses were performed using the R statistical computing environment [23]. Fitting of the model was done using the SPDE approach of the R-library INLA [24]. Maps of the study area were created using QGIS [25].

## Results

### Study population

**Fig. 2:**
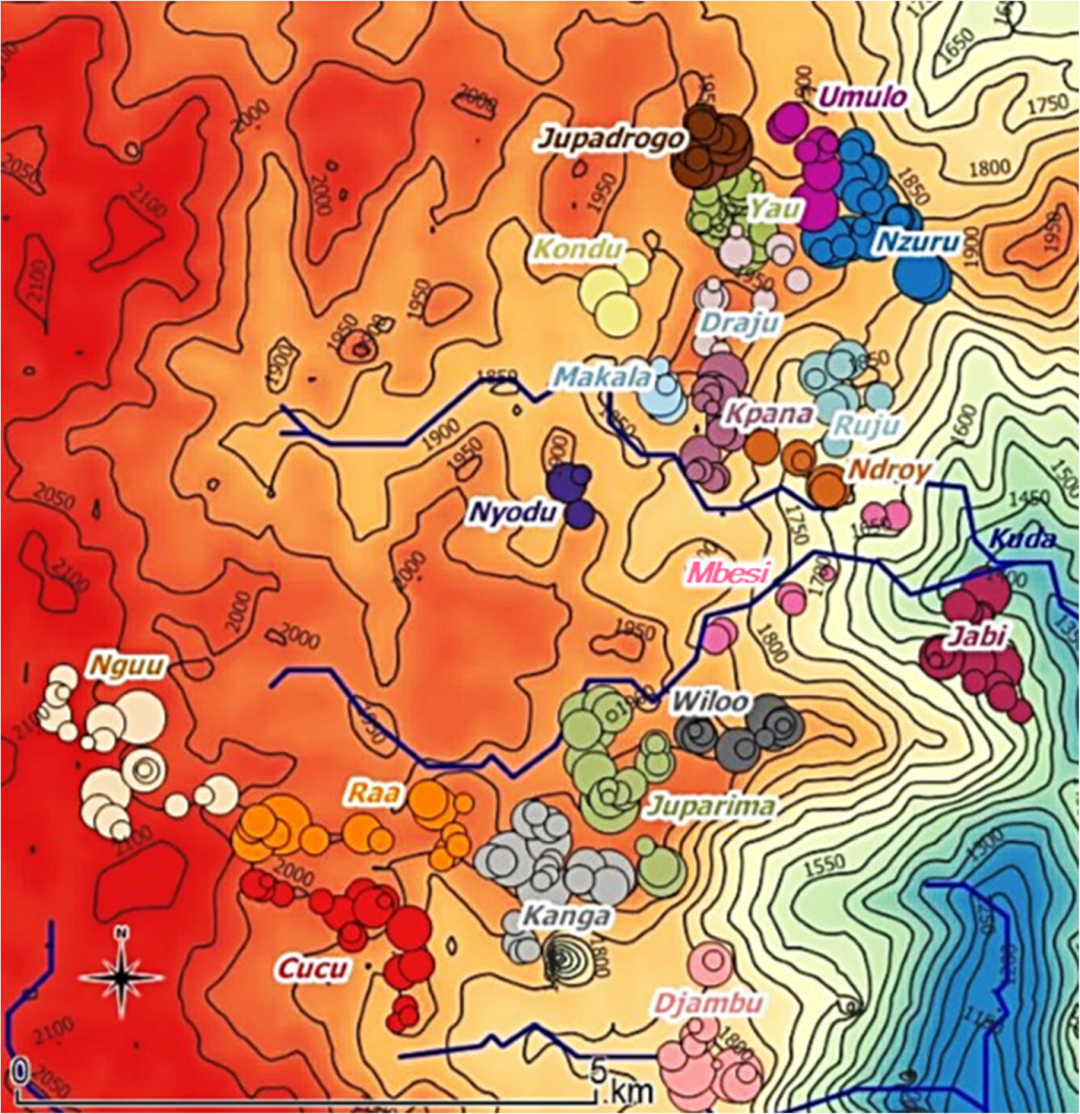
Distribution of sampled households by village. Topographical map showing the distribution of sampled households by village, with the size of the circles being proportional to the size of the households

A total of 256 households agreed to participate in the study, corresponding to 1403 individuals. Of these individuals, 14 (1%) refused to participate. Households were dispersed over 20 villages, covering two health areas: Draju (12 villages: Nzuru, Ruju, Kpana, Umulo, Makala, Ndroy, Mbesi, Yau, Draju, Kondu, Nyodu, Jupadrogo) and Kanga (eight villages: Kanga, Juparima, Jabi, Djambu, Cucu, Wiloo, Raa, Nguu) (Fig. 2). Two villages (1 in Draju health area, and 1 in Kanga health area) were not included, as no approval could be obtained from community leaders of these villages. Households consisted of a median of five members (interquartile range [IQR]: 4–7). Proxy responders (*n* = 1133) were fathers or husbands for 711 (62.8%) subjects, mothers or wives for 354 (31.2%) subjects and brothers, sisters, aunts and grandmothers for the remaining 68 (6.0%) subjects.

The median age of the study subjects was 15.0 years (IQR: 7–30); 34.5% were younger than 10 years; 51.3% were females (Table 1). The Kanga health area accounted for 53.6% of participants and the Draju health area for 46.4%. All participants belonged to the Alur ethnic group. Only 2.7% reported to have ever been treated with ivermectin.

**Table 1:** Characteristics of the study population

Characteristics	*N*	Percentage
Age groups		
0–9	472	34.5
10–19	374	27.3
20–29	170	12.4
30+	352	25.8
Total	1368	100.0
Gender		
Female	705	51.3
Total	1374	100.0
Health area		
Draju	645	46.4
Kanga	744	53.6
Total	1389	100.0
Ivermectin use		
Ever been treated with ivermectin	38	2.7
Total	1389	100.0

### Epilepsy prevalence and incidence

**Fig. 3:**
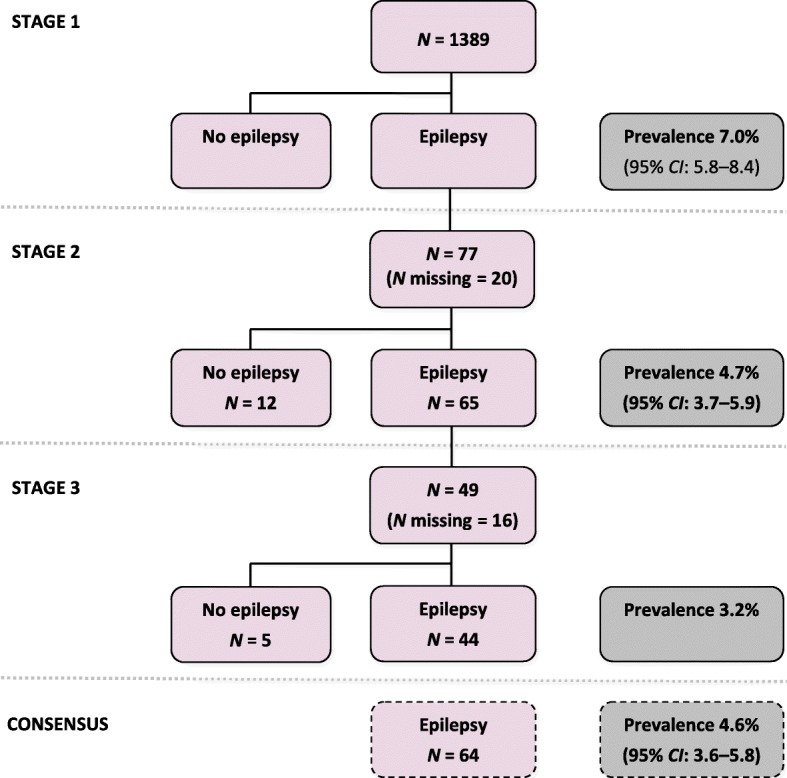
Three-stage process to identify persons with active epilepsy. In the first stage, all individuals of the selected households were screened for epilepsy by non-medical field workers. In the second and third stage, suspected cases of epilepsy were examined by non-specialist medical doctors, and by a neurologist, respectively. Those suspected cases of epilepsy who were not seen by the neurologist, were considered to be ‘true’ cases of epilepsy if they had at least three positive answers on the epilepsy screening questionnaire or had previously been told by a doctor to have epilepsy (review and consensus)

In stage 1, 1389 residents were screened for symptoms of epilepsy, of whom 97 (7.0%) (95% *CI*: 5.8–8.4) were suspected of having epilepsy (Fig. 3).

In stage 2, 77 (79.4%) of the 97 individuals with suspected epilepsy were examined by a general practitioner; 65 (84.4%) of them were confirmed to have epilepsy (prevalence 4.7%) (95% *CI*: 3.7–5.9). The reasons for ruling out epilepsy at this stage were febrile convulsions (*n* = 4), sleep disorders with night terrors (*n* = 1), vertigo (*n* = 1), headache (*n* = 1), misinterpretations of screening questions (*n* = 4) and epilepsy being no longer present (*n* = 1).

Only 49 (75.4%) of the 65 persons suspected of having epilepsy in stage 2 were seen by the neurologist (DM), of whom 44 (89.8%) were confirmed to have active epilepsy (prevalence 3.2%) (95% *CI*: 2.4–4.2). The reasons for ruling out epilepsy at this stage were febrile convulsions (*n* = 2), hyperventilation (*n* = 1), sleep disorders (*n* = 1) and only one episode of seizures (*n* = 1).

An additional 20 out of 36 people who were not seen by the neurologist, had at least 3 positive answers on the epilepsy screening questionnaire or had been previously told by a doctor to have epilepsy. Therefore, a total of 64 individuals were considered to be cases of epilepsy, corresponding to a prevalence of 4.6% (95% *CI*: 3.6–5.8).

The median age of persons with epilepsy was 17.0 years (IQR: 13–28) and 14 years (IQR: 7.0–30.0) for persons without epilepsy. The highest epilepsy prevalence (8.2%) was observed in the 20–29-year age group (Table 2).

**Table 2:** Prevalence of epilepsy by age group, gender, health area and ivermectin use

	Number of cases	Total sample	Epilepsy prevalence (%) (95% *CI*)
Age groups			
0–9	14	472	3.0 (1.8–4.9)
10–19	21	374	5.6 (3.7–8.4)
20–29	14	170	8.2 (5.0–13.4)
30+	15	352	4.3 (2.6–6.9)
Total	64	1368	4.7 (3.7–5.9)
Gender			
Male	30	669	4.5 (3.2–6.3)
Female	34	705	4.8 (3.5–6.7)
Total	64	1374	4.7 (3.7–5.9)
Health area			
Draju	35	645	5.4 (3.9–7.5)
Kanga	29	744	3.9 (2.7–5.5)
Total	64	1389	4.6 (3.6–5.8)
Ivermectin use			
Yes	3	38	7.9 (2.7–20.8)
No	61	1351	4.5 (3.5–5.8)
Total	64	1389	4.6 (3.6–5.8)

**Fig. 4:**
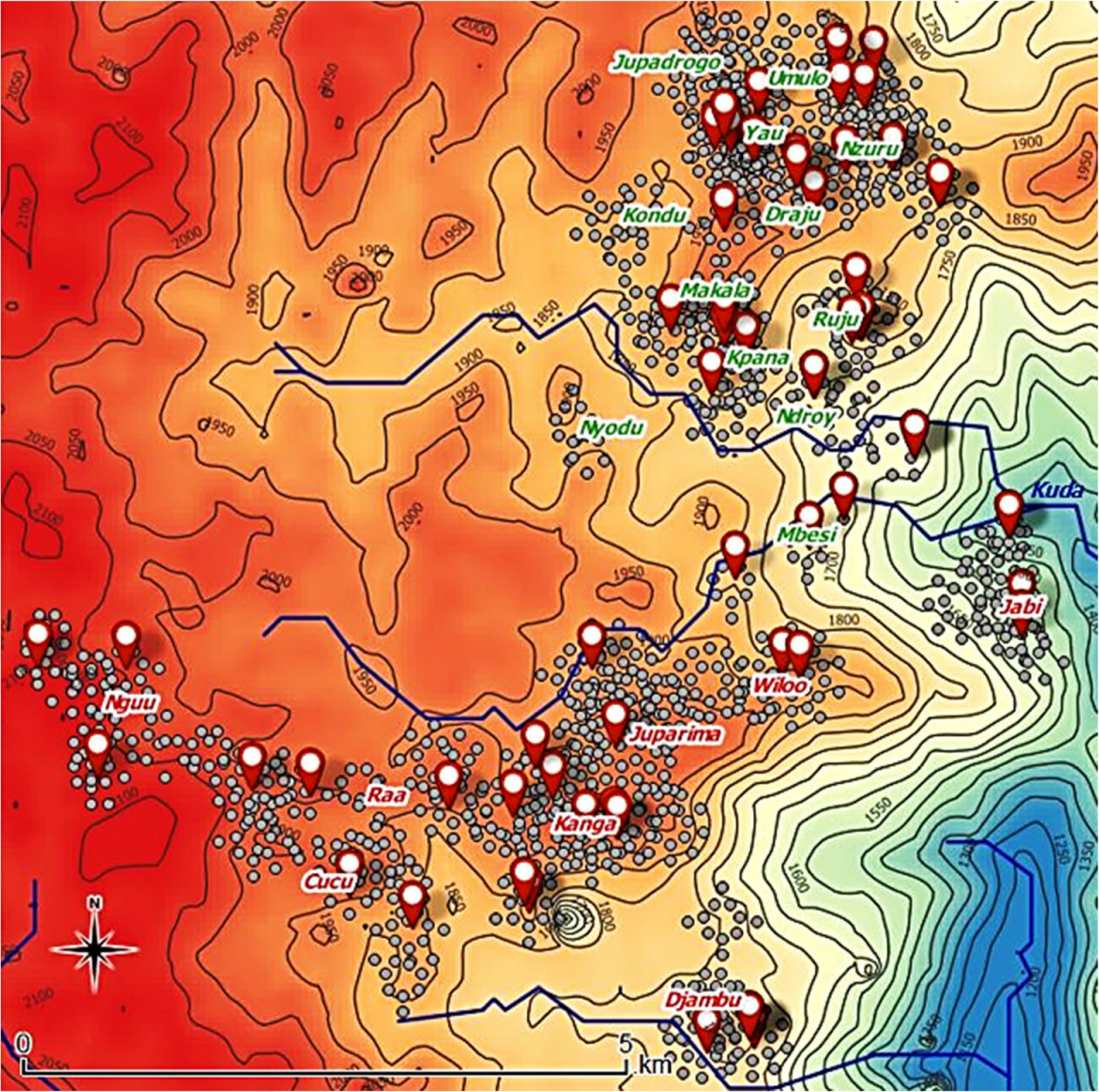
Distribution of people with epilepsy and people without epilepsy. Topographical map showing the distribution of people with epilepsy (red signs) and those without epilepsy (grey dots). Red-coloured village names refer to Kanga health area, whereas the green-coloured village names refer to Draju health area

**Fig. 5:**
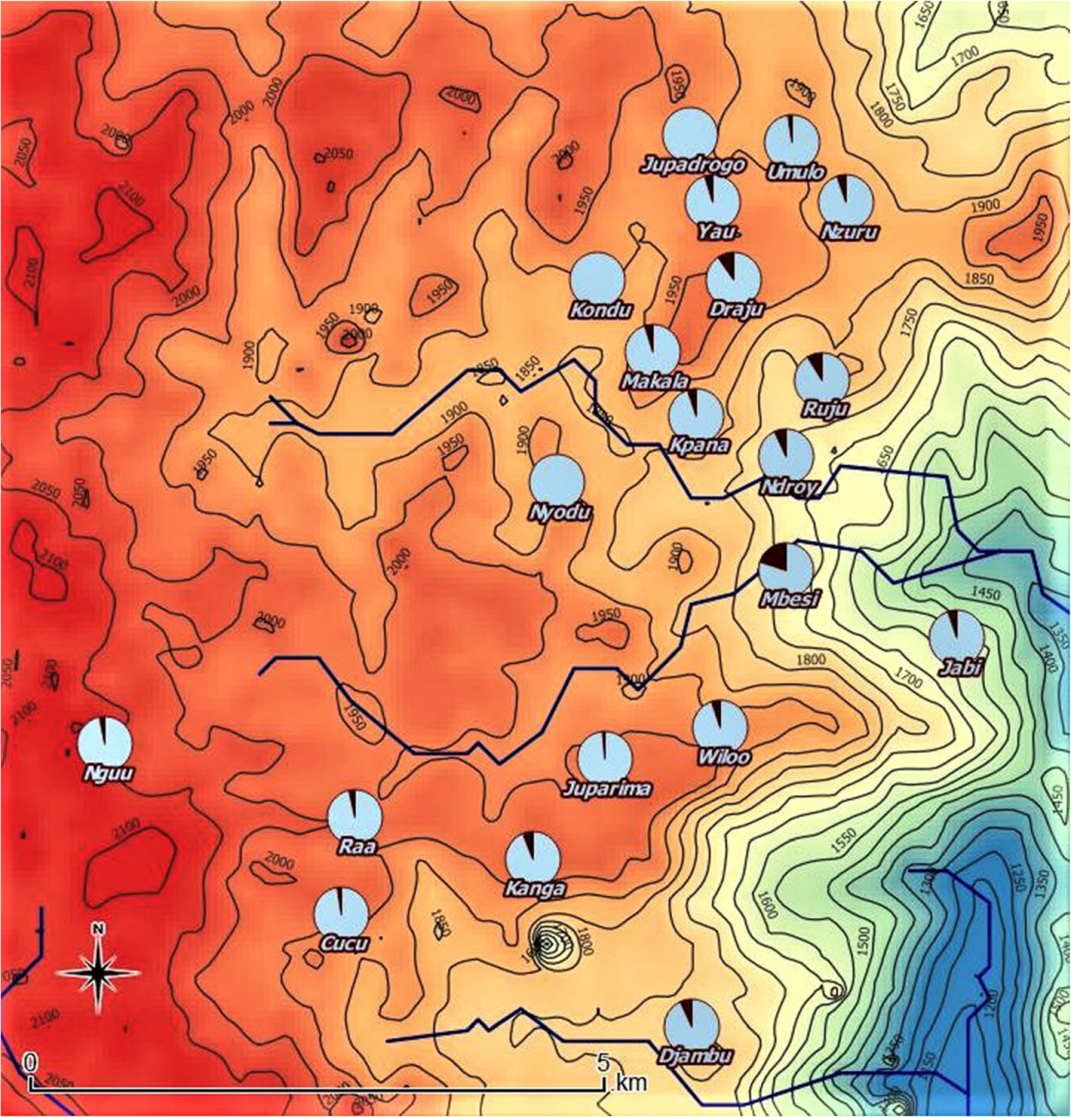
Epilepsy prevalence by village. Pie charts demonstrating epilepsy prevalence by village with dark-coloured slices being proportional to the number of people with epilepsy, and light-coloured slices being proportional to the number of people without epilepsy. See Additional file 2 for absolute numbers of people with epilepsy by village and corresponding village-specific epilepsy prevalence.

Similar prevalence figures were noted in females and males. Three out of 64 persons (4.7%) with epilepsy had ever been treated with ivermectin. Epilepsy prevalence was slightly, but not significantly, higher in Draju (5.4%) compared to Kanga (3.9%) (*P* = 0.20) (Fig. 4). The highest village-specific prevalence of epilepsy was noted in Mbesi (20.0%), followed by Draju (9.7%) and Ruju (8.9%) (Fig. 5) (Additional file 2).

In ten subjects, seizures appeared for the first time during the 12 months preceding the survey, corresponding to an annual incidence of epilepsy of 7.5 (95% *CI*: 3.8–13.4) per 1000 person-years. Age-specific incidence was 6.5 (95% *CI*: 1.7–17.8) per 1000 person-years for those below the age of 10, and 19.6 (95% *CI*: 8.6–38.8) per 1000 person-years for those aged 10 to 19 years. Gender specific incidence was 6.2 (95% *CI*: 2.0–15.1) per 1000 person-years for males, and 8.9 (95% *CI*: 3.6–18.5) per 1000 person-years for females. Epilepsy incidence was 9.8 (95% *CI*: 4.0–20.4) per 1000 person-years in Draju health area and 5.6 (95% *CI*: 1.8–13.5) per 1000 person-years in Kanga health area.

### Clinical characteristics of persons with epilepsy

**Fig. 6:**
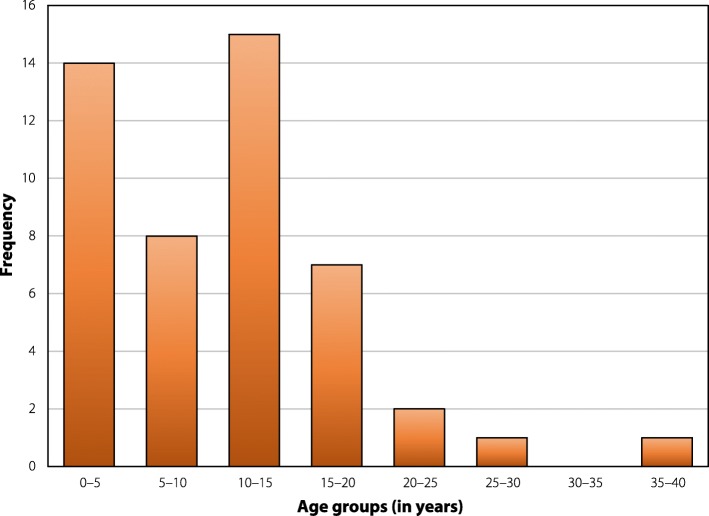
Age at onset of epilepsy. Bar chart showing the age at onset of epilepsy

While 64 individuals were considered to have active epilepsy, not all clinical data were available for these individuals. Age at onset of epilepsy was available for 48 subjects. The median age at onset of epilepsy was 10.0 years (IQR: 3.25–14.0); 15 (31.3%) were between ten and 15 years old (Fig. 6).

Of 48 subjects, 27 (56.3%) reported to have daily seizures. Generalized tonic-clonic seizures were the most common type of seizures (23/44, 52.3%). Absences were present in 4/44 individuals (9.1%). Although ‘head nodding’ was recorded in 15 (27.8%) of 54 subjects, a diagnosis of probable nodding syndrome could only be upheld in eight (14.8%) of 54 individuals with epilepsy. No specific trigger could be identified in 26/31 individuals (83.9%). Twenty (37.0%) of 54 subjects had other family members suffering from epilepsy.

Two persons (9.5%) had a low body mass index-for-age, eight (38.1%) presented with low height-for-age (Table 3). Four subjects (7.4%) showed no signs of sexual development. Visual impairment was noted in 24.1% of cases. Itching was present in 46.3%, pruritic papules in 13.0%. A leopard skin was observed in two individuals (3.7%), onchocercal nodules in another two. Ten subjects (18.5%) presented with burn scars, and nine (16.7%) with signs of tongue biting.

**Table 3:** Clinical assessment of persons with epilepsy

	*N*	%	Total number
Low BMI-for-age (< 2 *SD*)^a^	2	9.5	21
Low height-for-age (< 2 *SD*)^a^	8	38.1	21
Adolescents/adults with absence of signs of sexual development	4	7.4	54
Visual impairment	13	24.1	54
Skin conditions			54
Itching	25	46.3	
Pruritic papules	7	13.0	
Leopard skin	2	3.7	
Onchocercal nodules	2	3.7	
Burn scars	10	18.5	
Signs of tongue biting	9	16.7	

^a^*BMI* Body mass index, *SD* Standard deviation; based on WHO growth charts

### Potential risk factors for epilepsy

In 49 of 50 persons (96%) with epilepsy there was no history of birth trauma. In two (4.1%) of 49 persons with epilepsy, there was a history of prematurity, two (4.2%) had suffered from a head trauma in the past, one (2.0%) from meningitis and four (8.2%) had recovered from severe malaria; two (6%) had experienced febrile convulsions. Developmental delays before the onset of epilepsy were noted in seven (14.6%) of 48 individuals (not in table).

### Epilepsy treatment

Thirty-one (64.6%) of 48 subjects with confirmed epilepsy reported to have ever been treated with anti-epileptic drugs, mainly with phenobarbital (20/31, 64.5%) and phenytoin (4/31, 12.9%). Seven (22.6%) were treated continuously, 24 (77.4%) at irregular intervals; 20/48 (41.7%) reported to have ever used traditional medicine to treat epilepsy.

### *O. volvulus* exposure

OV16 tests were performed in 912 (65.7%) subjects (430 males and 482 females); 16 (1%) refused to have the test performed, 97 (7%) were younger than 3 years and 364 (26%) were absent when their household was visited. The following results only relate to those persons for whom OV16 test results were available.

OV16 test results were positive in 278 (30.5%) (95% *CI*: 27.6–33.6). The proportion of positive test results increased with increasing age groups until the age of 40 (Table 4). OV16 tests were positive in 18 (58.1%) of 31 people who had ever been treated with ivermectin, and in 260 (29.5%) of 881 people who had never been treated with ivermectin. Twenty-five (46.3%) of the 54 persons with epilepsy in whom OV16 tests were performed, were positive, compared to 253 (29.5%) of 858 subjects without epilepsy (*P* = 0.014).

**Table 4:** Percentage of individuals with a positive OV16 antibody test by age category, gender, health area, ivermectin use and diagnosis of epilepsy

	Number of OV16^+^	Number of OV16^−^	Percentage of OV16^+^ (95% *CI*)
Age groups			
0–9	12	265	4.3 (2.5–7.4)
10–19	51	196	20.7 (16.1–26.1)
20–29	52	62	45.6 (36.8–54.8)
30–39	55	30	64.7 (54.1–74.0)
40–49	46	33	58.2 (47.2–68.5)
50+	62	48	56.4 (47.0–65.3)
Gender			
Male	125	305	29.1 (25.0–33.5)
Female	153	329	31.7 (27.8–36.0)
Health area			
Draju	155	278	35.8 (31.4–40.4)
Kanga	123	356	25.7 (22.0–29.8)
Ivermectin use			
Yes	18	13	58.1 (40.8–73.6)
No	260	621	29.5 (26.6–32.6)
Epilepsy			
Yes	25	29	46.3 (33.7–59.4)
No	253	605	29.5 (26.5–32.6)

*OV16*^*+*^ OV16 Positive, *OV16*^*−*^ OV16 negative

**Fig. 7:**
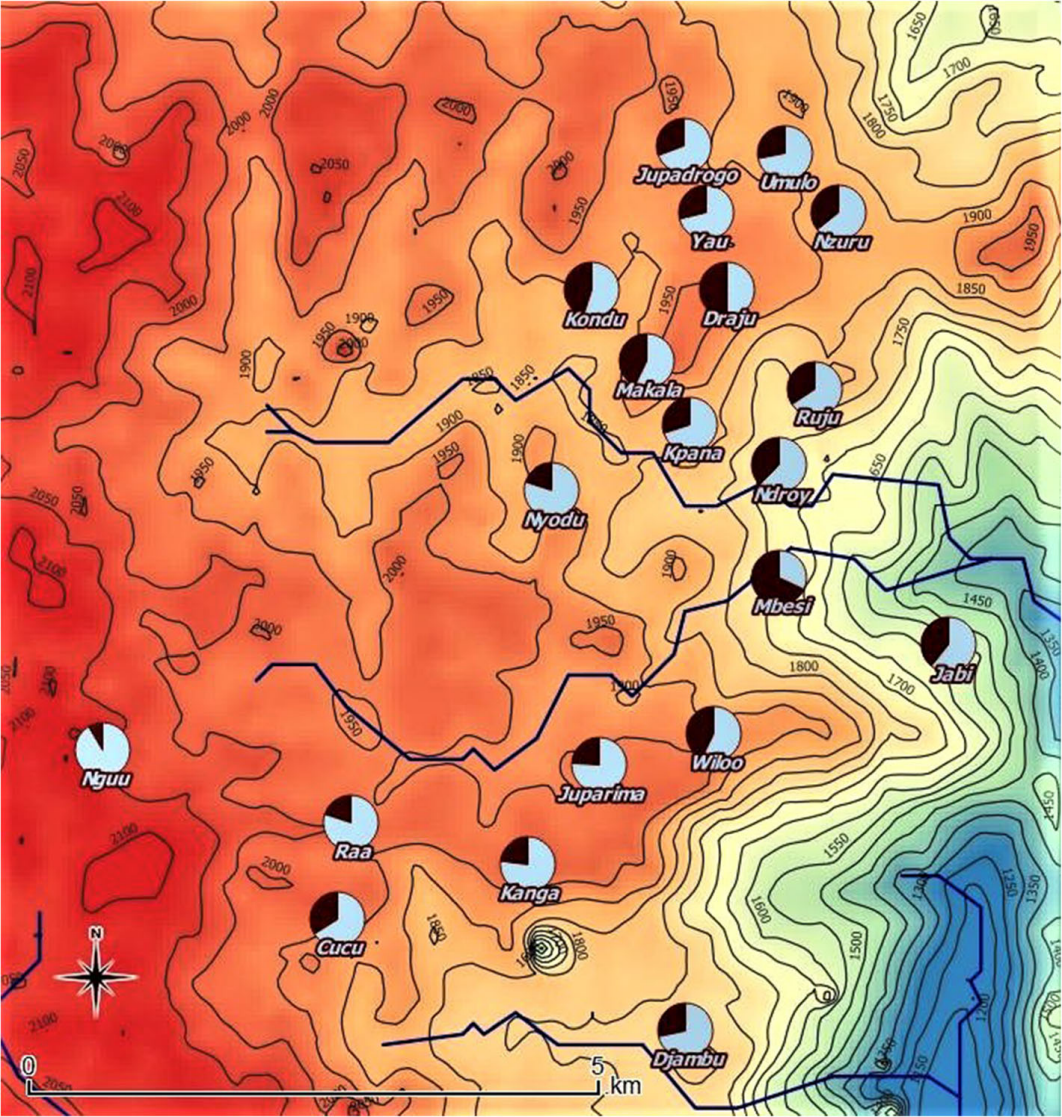
OV16 seroprevalence by village. Pie charts demonstrating OV16 seroprevalence by village with dark-coloured slices being proportional to the number of people with positive OV16 test results, and light-coloured slices being proportional to the number of people with negative OV16 test results. See Additional file 3 for absolute numbers of people with positive and negative OV16 test results and corresponding percentages of individuals with a positive OV16 antibody test result by village

The seroprevalence of OV16 ranged from 25.7 to 35.8% in the different health areas, and from 8.6 to 68.0% in the sampled villages (Fig. 7) (Additional file 3). The highest proportion of positive test results were found in the village with the highest prevalence of epilepsy (Mbesi).

### Spatial analysis

The results from the spatial analysis (adjusted for age, gender, ivermectin use, household membership and location of residence) showed a significant association between epilepsy and exposure to *O. volvulus* with an adjusted odds ratio (a*OR*) of 3.19 (95% *CI:* 1.63–5.64, *P* = 0.001). The results also showed that OV16 positivity, but not the presence of epilepsy, was significantly associated with lower altitude. For every 1000 m increase in altitude, the odds of being OV16 positive was approximately 90% lower than the odds of being OV16 negative (a*OR* = 0.09, 95% *CI*: 0.01–0.31).

A significant neighbour effect was noted for both epilepsy and OV16 seropositivity. The odds of being positive for epilepsy increased by more than 100% for each additional case of epilepsy within the neighbourhood (250 m distance) (a*OR* = 2.19, 95% *CI*: 1.69–2.82). The odds of being OV16 positive increased by approximately 15% for each additional OV16 positive case within the neighbourhood (250 m distance) (a*OR* = 1.15, 95% *CI*: 1.09–1.23). Spatial aggregation/autocorrelation of cases was found within approximately 400 m for epilepsy and 300 m for OV16. Outside of this range, the correlation became negligible.

## Discussion

This study confirms the high epilepsy prevalence (4.6%) and incidence (7.5 per 1000 person-years) in two health areas of the Logo health zone (Ituri Province), although this is lower than the prevalence of 6% reported in a survey in Draju Village in 2015 [11]. The latter may reflect an overestimation of epilepsy prevalence, most likely due to the rapid assessment methodology used in 2015. In the current study, persons with only one episode of seizures were not considered as persons with epilepsy, because there was no underlying pathology with a proven 60% chance to experience new episodes of seizures. The 2014 ILAE guidelines define epilepsy as two episodes of unprovoked seizures 24 h apart or one episode of seizures with a proven 60% chance to experience new episodes of seizures (e.g., after a stroke, infection of the central nervous system or trauma) [18]. However, it should be investigated whether a first episode of seizures occurring in a previously healthy individual between three and 18 years of age, living in an onchocerciasis endemic area, should also be considered as epilepsy because of the high likelihood of recurrent seizures.

Furthermore, nodding syndrome was suspected in eight out of 54 individuals with epilepsy. This is the first time that nodding type of seizures has been reported in the DRC. Thus far, one of the main arguments against *O. volvulus* as the trigger of nodding syndrome, is that this disease does not exist in onchocerciasis endemic regions outside of Uganda, South Sudan and Tanzania. This study suggests that nodding syndrome may be underdiagnosed in onchocerciasis endemic regions, simply because it has never been investigated. During a recent epilepsy prevalence study in the Mbam Valley, an onchocerciasis endemic region in Cameroon, persons presenting with nodding type of seizures were observed as well [26]. Nevertheless, a diagnosis of nodding syndrome was only made by history taking and therefore did not meet the criteria of confirmed nodding syndrome, as proposed by the World Health Organization (WHO) [27]. However, the presence of stunted adolescents/adults with epilepsy without external signs of sexual development (clinical characteristics of Nakalanga syndrome [28]) in villages in the Logo health zone suggest that nodding syndrome is also present in this area. Symptoms of Nakalanga and nodding syndrome overlap and both syndromes are only observed in onchocerciasis endemic regions, together with a high prevalence of other forms of epilepsy [29].

Spatial analysis showed that OV16 positivity, but not the presence of epilepsy, was significantly associated with lower altitude (closer to the river Kuda). The explanation for this finding could be the relatively low number of persons (with onchocerciasis associated epilepsy [OAE]) sampled at high altitude in this study.

Our study has several limitations. A three-stage approach was used to identify cases with epilepsy, but attrition between survey stages was high. A similar problem was reported by Ngugi et al., during epilepsy surveys in 5 demographic surveillance sites in Africa [12]. They adjusted their estimates for attrition in their analyses using multiple imputation and sensitivity adjustments (by dividing prevalence estimates by the estimated sensitivity). For multiple imputation to be valid, data must be ‘missing at random’ (i.e. the probability of missing data on a variable does not depend on itself). However, we were not convinced that our missing data, related to epilepsy prevalence, were ‘missing at random’. We hypothesized that people with suspected epilepsy at the stage of screening may not have made it to the subsequent stages of validation and/or confirmation because of reasons related to their epilepsy. Furthermore, sensitivity adjustments could not be applied to our data, as our study was not designed to validate the three-stage methodology. Our study did not include a ‘golden standard’ against which the three-stage methodology could be evaluated. Moreover, those not suspected of epilepsy at the stage of screening were not seen by a trained health professional. Therefore, we could not know whether these ‘negative’ cases represented ‘false negative’ cases, a prerequisite to calculate sensitivity.

Furthermore, the epilepsy screening questionnaire used in this study was only validated in Mauritania, but not locally. In addition, laboratory investigations and neuro-imaging to identify alternative causes of epilepsy, such as neurocysticercosis, and video-electro-encephalograms to document the type of seizures, were not performed. However, neurocysticercosis does not seem to be a frequent cause of epilepsy in this area, because the onset of this form of epilepsy is generally after the age of 20 [30, 31]. Moreover, in a case-control study performed in the area, only two of 218 participants tested positive for the presence of *Taenia Solium* antigens (Colebunders R, unpublished).

Bias may have been introduced in our study in several ways. As epilepsy was only reported, but not observed, we cannot exclude the possibility of response bias. Age at epilepsy onset, and resulting incidence estimates, may have been prone to recall bias. Additionally, interviewers in the validation and confirmation stage were aware of the potential epileptic status of participants, which could have introduced information bias. Moreover, detailed clinical examinations were only performed in subjects with suspected epilepsy. Although epilepsy prevalence was calculated at village level, little inference can be made from these estimates, due to the low number of individuals sampled from each village.

Another point that needs to be addressed, is the low, but still ongoing transmission of *O. volvulus*, as indicated by the low percentage of OV16 positive children. At repeated visits to the area, it was difficult to find actively biting anthropophilic blackflies. Interviews with local people revealed that in recent years, exposure to blackflies has decreased. The current *O. volvulus* transmission zones seem to be limited to recently identified valley bottoms with riverine gallery forest or banana tree edges, located within flight range of blackflies. We hypothesize that deforestation in the area over the last 30 years has caused a decrease in the blackfly population, because the local *O. volvulus* vector is forest dependent (photophobic, cold weather species).

However, given the ongoing onchocerciasis transmission and the high prevalence of epilepsy in the health areas included in this study, a CDTI programme needs to be established, together with a decentralised system to treat persons with epilepsy. This will require task shifting of the clinical management of epilepsy to lower cadres and an increase of access to anti-epileptic treatment. In collaboration with Malteser International [32], which has been working in the eastern provinces of the DRC since 1996, we initiated such a programme in the villages of Draju and Kanga in October 2017.

## Conclusions

A high prevalence of epilepsy and a significant association between epilepsy and exposure to *O. volvulus* were observed in the rural health areas of Draju and Kanga (Logo health zone, Ituri Province). There is an urgent need to implement a CDTI programme and to scale up an epilepsy treatment and care programme.

## Additional files

Additional file 1: Multilingual abstract in the five official working languages of the United Nations. (PDF 248 kb)

Additional file 2: Village-specific prevalence of epilepsy. (PDF 11 kb)

Additional file 3: Percentage of individuals with a positive OV16 antibody test result by village. (PDF 11 kb)

## Abbreviations

BMI: Body mass index
CDTI: Community-directed treatment with ivermectin
*CI*: Confidence interval
DRC: Democratic Republic of Congo
HDI: Human development index
ILAE: International League Against Epilepsy
OAE: Onchocerciasis associated epilepsy
*OR*: Odds ratio
REMO: Rapid epidemiological mapping of onchocerciasis
*SD*: Standard deviation
WHO: World Health Organization
