# Supplementary material for: High prevalence of epilepsy in onchocerciasis endemic health areas in Democratic Republic of the Congo
Source: Infect Dis Poverty. 2018 Aug 1;7:68. doi: 10.1186/s40249-018-0452-1 (PMC6069757; doi:10.1186/s40249-018-0452-1)
Supplement: Supplementary file 1 — Multilingual abstract in the five official working languages of the United Nations. (PDF 248 kb) [file 40249_2018_452_MOESM1_ESM.pdf]

Translation of the abstract into the five official working languages of the United Nations

ارتفاع معدل انتشار داء الصرع في المناطق الصحية الموبوءة بالعمى النهري في إقليم إيتوري، بجمهورية الكونغو الديموقراطية.

قدمه: إيفي لينايرتس , وميشال ماندرو، وديبي موكندي، وباتريك سوكربوك، وحسيني دولو، وديوغرايتيس وونيا روسي، وفرانسوا نجيف، وكليافيإينسوي-موسورو، وآن لاوديزويت، وإن هوتوربيكس وروبرت كولبندرز

الملخص
المعلومات الأساسية: لقد لوحُظ ارتفاع معدل انتشار داء الصرع في العديد من المناطق الموبوءة بالعمى النهري. تهدف هذه الدراسة إلى تقدير معدل انتشار داء الصرع النشط واحتمالات التعرض لعدوى دودة كلابية الذنب المتلوية بين سكان الريف في إقليم إيتوري،بجمهورية الكونغو الديموقراطية.
الأساليب: في شهر أغسطس ٢٠١٧م، أُجريت دراسة شاملة لعدة قطاعات وقائمة على المجتمع في منطقة موبوءة بالعمى النهري في منطقة لوغو الصحية الريفية، بإقليم إيتوري لقد أُخذت عينات عشوائية من الأسر التي تعيش ضمن حدود كل منطقتين صحيتين بالجوار. فبغرض التعرف على الأشخاص المصابين بالصرع، أُستخدم نهج يتألف من ثلاثة مراحل. في المرحلة الأولى، جرى فحص داء الصرع على كافة الأفراد من الأسر المُختارة من جانب العاملين الميدانيين غير الطبيين باستخدام استبيان مُصدق عليه يتألف من ٥ عناصر. في المرحلة الثانية والثالثة، خضعت الحالات التي يشتبه إصابتها بالصرع للفحص على يد الأطباء غير المتخصصين، وطبيب الأمراض العصبية، على التوالي. حُددت حالة للإصابة بداء الصرع وفقاً للمبادئ التوجيهية للعام ٢٠١٤ م التابعة للعصبة الدولية لمكافحة داء الصرع. تم تقييم احتمالات التعرض لدودة كلابية الذنب المتلويةباجراء الفحوص للكشف عن الأجسام المضادة (IgG4) لمستضد دودة كلابية الذنب المتلوية(الفحص التشخيصي السريع OV16,SD Bioline,Inc.) على الأشخاص بعمر ثلاث سنوات فما فوق.
النتائج: من بين 1389 مشارك من المشمولين في الدراسة الاستقصائية، 64 منهم اشتبه بإصابتهم بداء الصرع النشط (معدل الانتشار=4.6%) [95% فاصل الثقة (فاصل الثقة): 3.6–5.8]. لُوحظ التقدير الأعلى لمعدل انتشار داء الصرع بين فئة عمرية محددة في الذين تتراوح أعمارهم من ٢٠ وحتى ٢٩ عاماً بنسبة (8.2%). وكان متوسط العمر الذي يبدأ فيه داء الصرع بالظهور هو ١٠ سنوات، مع بلوغه الذروة في الفئة العمرية التي تتراوح أعمارهم من ١٠ وحتى ١٥ عاماً. كانت نتائج فحص OV16 متوفرة لعدد 912 مشاركاً، من بينهم 30.5% كانت نتيجة فحصهم (95% فاصل الثقة: 27.6–33.6) إيجابية. يتراوح معدل انتشار الإصابة بعدوى كلابية الذنب المتلوية عبر فحص OV16 في قرية ما من 8.6 إلى 68.0%. ووُجِدَ أن أعلى انتشار لنتائج تحليل الدم الإيجابية كان في قرية ذات معدل الانتشار الأعلى للصرع. فبعد مراعاة العمر، والجنس و استخدام الآيفرمِكتين، لوحُظ وجود علاقة مهمة بين احتمالية التعرض للعمى النهري والصرع (نسبة الأرجحية المُعدلة= 3.19, 95% فاصل الثقة: 1.63–5.64) (القيمة الاحتمالية< 0.001).
الاستنتاجات: لُوحظ معدل انتشار عالٍ لداء الصرع ووجود علاقة مهمة بين الصرع واحتمالية التعرض لدودة كلابية الذنب المتلوية في سكان منطقة لوغو الصحية بإقليم إيتوري، بجمهورية الكونغو الديموقراطية.

Translated from English version into Arabic by Bashaier Allam and Aseel Abdulrahman, through

TRANSLATORS
WITHOUT BORDERS

刚果民主共和国健康区中盘尾丝虫病流行率与癫痫患病率显著相关

Evy Lenaerts, Michel Mandro, Deby Mukendi, Patrick Suykerbuyk, Housseini Dolo, Deogratias Wonya'Rossi, Françoise Ngave, Chellafe Ensoy-Musoro, Anne Laudisoit, An Hotterbeekx and Robert Colebunders

摘要

引言：许多盘尾丝虫病流行地区的癫痫发病率也较高。本研究旨在评估刚果民主共和国伊图里省的农村人口中活动性癫痫的流行率和旋盘尾丝虫感染的暴露情况。

方法：2016 年 8 月，我们在伊图里省 Logo 农村健康区域的盘尾丝虫病流行区开展了一项以社区为基础的横断面研究。在两个相邻健康区域内的住户中进行随机抽样。研究人员采用了三阶段法确认癫痫患者。在第一个阶段，非医疗工作者使用一份经过验证的 5 项问卷对纳入家庭中的所有个体进行筛查。在第二和第三阶段，非专科医生以及神经科医师分别检查癫痫疑似病例。根据 2014 年国际抗癫痫协会(ILAE)的指南对确认癫痫病例。在 3 岁及以上的个体中，通过 IgG4 抗体测试旋盘尾丝虫抗原来评估其暴露情况 (OV16 快速检测，SD Bioline, Inc.)。

结果：在 1389 名参与调查的参与者中，64 人被诊断患有活动性癫痫(患病率= 4.6%)[95%置信区间(*CI*): 3.6–5.8]。20 至 29 岁的人群中年龄特异性癫痫患病率较高(8.2%)。癫痫发作的年龄中位数为 10 岁，在 10 至 15 岁年龄组中癫痫发生率最高。研究人员对 912 名参与者进行 OV16 检测，发现其中 30.5% (95% CI: 27.6 33.6)为呈阳性。在一个村庄中，OV16 阳性率在 8.6 到 68.0%之间。在癫痫发病率最高的村庄中，阳性血清学结果也最高。在校正年龄、性别和伊维菌素使用后，我们发现盘尾丝虫病和癫痫存在显著相关性(aOR = 3.19，95% *CI*: 1.63–5.64) ($P < 0.001$)。

结论：在刚果民主共和国伊图里省的 Logo 健康区中癫痫的发病率较高，癫痫和旋盘尾丝虫的暴露之间存在显著相关性。

Translated from English version into Chinese by Xin-Yu Feng, edited by Jin Chen

Forte prévalence de l’épilepsie dans les régions d’onchocercose endémique de la région de l’Ituri, en République démocratique du Congo

Evy Lenaerts, Michel Mandro, Deby Mukendi, Patrick Suykerbuyk, Housseini Dolo, Deogratias Wonya’Rossi, Françoise Ngave, Chellafe Ensoy-Musoro, Anne Laudisoit, An Hotterbeekx et Robert Colebunders

Résumé

Contexte : Une prévalence élevée de l’épilepsie a été observée dans de nombreuses régions d’onchocercose endémique. Le but de la présente étude est d’estimer la prévalence de l’épilepsie active et de l’exposition au parasite *Onchocerca volvulus* (*O. volvulus*) dans une population rurale de la province de l’Ituri, en République démocratique du Congo.

Méthodes : Une étude transversale communautaire a été menée en août 2016 dans une région d’onchocercose endémique, dans la zone sanitaire rurale de Logo (province de l’Ituri). Les foyers de 2 aires sanitaires voisines ont été inclus par échantillonnage aléatoire. Une approche en trois temps a été employée pour identifier les personnes atteintes d’épilepsie : dans un premier temps, des agents de terrain non médicaux ont dépisté les cas d’épilepsie parmi toutes les personnes des foyers sélectionnés à l’aide d’un questionnaire en cinq points. Les cas suspects d’épilepsie ont été

examinés par un médecin non spécialiste dans un deuxième temps puis, dans un troisième temps, par un neurologue. L'épilepsie a été définie selon les recommandations de 2014 de la Ligue internationale contre l'épilepsie (ILAE). L'exposition à *O. volvulus* a été évaluée chez tous les sujets âgés de 3 ans et plus par la recherche des anticorps IgG visant un antigène du parasite (OV16 Rapid Test, SD Bioline, Inc.).

Résultats : Sur les 1389 participants inclus dans l'étude, 64 ont été considérés comme porteurs d'une épilepsie active (prévalence = 4,6 % ; intervalle de confiance (IC) à 95 % de 3,6 à 5,8). L'estimation la plus élevée de la prévalence en fonction de l'âge a été observée chez les sujets âgés de 20 à 29 ans (8,2 %). L'âge médian de manifestation de l'épilepsie était de 10 ans, avec une incidence maximale dans le groupe des 10-15 ans. Les résultats du test OV16 ont été obtenus pour 912 participants, dont 30,5 % (IC à 95 % de 27,6 à 33,6) étaient positifs. La prévalence de la positivité à OV16 dans un même village variait entre 8,6 et 68,0 %. La prévalence la plus élevée des résultats de sérologie positifs s'observait dans le village ayant la plus forte prévalence d'épilepsie. Après ajustement en fonction de l'âge, du sexe et de la prise d'ivermectine, nous avons observé une association significative entre l'exposition à l'onchocercose et l'épilepsie (rapport de cotes ajusté = 3,19, IC à 95 % de 1,63 à 5,64 ; $P < 0,001$).

Conclusions : Une prévalence élevée de l'épilepsie et une association significative entre celle-ci et l'exposition à *O. volvulus* ont été observées dans la population de la zone sanitaire de Logo, dans la province de l'Ituri en République démocratique du Congo.

Translated from English version into French by Suzanne Assenat and Isabelle2018, through

TRANSLATORS
WITHOUT BORDERS

Высокая распространённость эпилепсии в эндемичных очагах онхоцеркоза в провинции Итури Демократической Республики Конго.

Эви Ленартс, Михель Мандро, Деби Мукенди, Патрик Сёйкербёйк, Хуссейни Доло, Деогратиас Вониа'Росси, Франсуаза Нгаве, Челлафе Энсой-Мусоро, Анн Лаудисойт, Ан Хоттербекс и Роберт Коулбандерс

Реферат

Справочная информация: Во многих эндемичных очагах онхоцеркоза наблюдается высокая распространённость эпилепсии. Целью настоящего исследования является оценка распространённости активной формы эпилепсии, а также воздействия инфекции *Onchocerca volvulus* (*O. volvulus*) на сельское население провинции Итури Демократической Республики Конго.

Методы: В августе 2016 года в эндемичном очаге онхоцеркоза, расположенном в сельской санитарной зоне Лого провинции Итури, было проведено перекрёстное исследование на уровне общин. Методом случайной выборки были отобраны домохозяйства, находящиеся в пределах 2 соседних медицинских округов. Выявление лиц, страдающих эпилепсией,

производилось с помощью трёхэтапного алгоритма. На первом этапе, используя утверждённую анкету из пяти вопросов, немедицинские сотрудники на местах произвели обследование всех проживающих в отобранных домохозяйствах лиц на наличие эпилепсии. На втором и третьем этапах больные с подозрением на эпилепсию прошли обследование у врачей общего профиля и неврологов соответственно. Выявление случаев эпилепсии производилось в соответствии с изданными в 2014 году рекомендациями Международной лиги по борьбе с эпилепсией (ILAE). Оценка воздействия *O. volvulus* производилась тестированием на присутствие антител IgG4 к антигену *O. volvulus* (экспресс-тест OV16, SD Bioline, Inc.) у больных в возрасте 3 лет и старше.

Результаты: Из 1389 участников, включённых в исследование, 64 человека были оценены как страдающие активной формой эпилепсии (показатель распространённости = 4,6%) [доверительный интервал 95% (*CI*): 3,6–5,8]. Наиболее высокий показатель распространённости эпилепсии по возрастному признаку наблюдался в возрастной группе от 20 до 29 лет (8,2%). Медианный возраст возникновения заболевания составил 10 лет, при этом пик заболеваемости эпилепсией пришёлся на возрастную группу от 10 до 15 лет. Результаты теста OV16 были получены по 912 участникам, 30,5% из которых (95% *CI*: 27,6–33,6) дали положительную реакцию. Распространённость положительности теста OV16 в деревне колебалась в пределах от 8,6 до 68,0%. Наиболее высокий показатель распространённости позитивных результатов серологических анализов был обнаружен в деревне с наиболее высокой распространённостью эпилепсии. После внесения корректив по возрасту, полу и применению ивермектина наблюдалась значительная связь между воздействием онхоцеркоза и эпилепсией (скорректированное соотношение шансов = 3,19, 95% *CI*: 1,63–5,64) ($P < 0,001$).

Выводы: Среди населения санитарной зоны Лого в провинции Итури Демократической республики Конго наблюдалась высокая распространённость эпилепсии, а также значительная связь между эпилепсией и воздействием *O. volvulus* .

Translated from English version into Russian by Liudmila Tomanek and Ekaterina Rugg , through

TRANSLATORS
WITHOUT BORDERS

La alta prevalencia de epilepsia en zonas sanitarias en las que la oncocercosis se ha catalogado como enfermedad endémica dentro de la provincia de Ituri, en la República Democrática del Congo.

Evy Lenaerts, Michel Mandro, Deby Mukendi, Patrick Suykerbuyk, Housseini Dolo, Deogratias Wonya'Rossi, Françoise Ngave, Chellafe Ensoy-Musoro, Anne Laudisoit, An Hotterbeekx y Robert Colebunders

Resumen

Antecedentes: Se ha observado una alta prevalencia de epilepsia en regiones en las que la oncocercosis está catalogada como enfermedad endémica. Este estudio tiene como objetivo determinar la prevalencia de epilepsia activa, así como la exposición a la infección por *Onchocerca volvulus* (*O. volvulus*) en una población rural de la provincia de Ituri, en la República Democrática del Congo.
Metodología: En agosto de 2016, se llevó a cabo un estudio comunitario transversal en la zona sanitaria rural de Logo, en la provincia de Ituri, donde la oncocercosis es endémica. Se tomaron muestras de forma aleatoria en domicilios pertenecientes a dos zonas sanitarias vecinas. Para identificar a aquellas personas con epilepsia, se utilizó un proceso que consta de tres fases. En la primera fase, todos los individuos de los domicilios fueron examinados para diagnosticar la posible epilepsia por personal de campo no médico mediante un cuestionario validado con 5 puntos. En la segunda y tercera fase, los individuos de los que se pensaba que podían padecer epilepsia fueron examinados por personal médico no especialista y por un neurólogo, respectivamente. Se definió un caso de epilepsia según las directrices de la Liga Internacional contra la Epilepsia (ILAE) de 2014. La exposición a *O. volvulus* se evaluó mediante la prueba para detectar anticuerpos IgG4 contra el antígeno de *O. volvulus* (Prueba Rápida OV16, SD Bioline, Inc.) en individuos con edades a partir de los 3 años.
Resultados: De un total de 1389 participantes incluidos en la encuesta, se consideró que 64 tenían epilepsia activa (prevalencia = 4,6 %) [intervalo de confianza (*IC*) del 95 %: 3,6 - 5,8]. La estimación más alta de la prevalencia de epilepsia por edad se observó en personas de 20 a 29 años (8,2 %). La mediana de edad en la que se manifiesta la epilepsia son los 10 años, con una incidencia máxima de la misma en el grupo de edad comprendido entre los 10 y los 15 años. Los resultados de la prueba OV16 estaban disponibles para 912 participantes, de los cuales el 30,5 % arrojaron resultados positivos (con un 95 % *de IC*: 27,6 - 33,6) La prevalencia de OV16 positivo en un pueblo fluctuó del 8,6 al 68 %. La prevalencia más alta de resultados serológicos positivos se encontró en el pueblo con la mayor prevalencia de epilepsia. Después de ajustar por edad, sexo y uso de ivermectina, se observó una asociación significativa entre la exposición a la oncocercosis y la epilepsia (cociente de posibilidades ajustada = 3,19 con un *IC*del 95 %: 1,63 - 5,64)(*P* < 0,001).
Conclusiones: Se observó una alta prevalencia de epilepsia, así como una asociación entre la epilepsia y la exposición al*O. volvulus* , en la población de la zona sanitaria de Logo, en la provincia de Ituri de la República Democrática del Congo.

Translated from English version into Spanish by Tamara_jj and Noelia Bernárdez, through

TRANSLATORS
WITHOUT BORDERS
