# Supplementary material for: High prevalence of epilepsy in onchocerciasis endemic health areas in Democratic Republic of the Congo
Source: Infect Dis Poverty. 2018 Aug 1;7:68. doi: 10.1186/s40249-018-0452-1 (PMC6069757; doi:10.1186/s40249-018-0452-1)
Supplement: Supplementary file 2 — Village-specific prevalence of epilepsy. (PDF 11 kb) [file 40249_2018_452_MOESM2_ESM.pdf]

Additional file 1. Village-specific prevalence of epilepsy

Health area	Village	N of cases	Total sample	Prevalence (%) [95% CI]
**Draju**	Nzuru	7	132	5.3 [2.6-10.5]
	Ruju	4	45	8.9 [3.5-20.7]
	Kpana	4	78	5.1 [2.0-12.5]
	Umulo	1	41	2.4 [0.4-12.6]
	Makala	2	42	4.8 [1.3-15.8]
	Ndroy	2	29	6.9 [1.9-22.0]
	Mbesi	6	30	20.0 [9.5-37.3]
	Yau	5	105	4.8 [2.1-10.7]
	Draju	3	31	9.7 [3.3-24.9]
	Kondu	0	26	0.0 [0.0-12.9]
	Nyodu	0	14	0.0 [0.0-21.5]
	Jupadrogo	0	72	0.0 [0.0-5.1]
**Kanga**	Kanga	8	132	6.1 [3.1-11.5]
	Juparima	2	141	1.4 [0.4-5.0]
	Jabi	4	76	5.3 [2.1-12.8]
	Djambu	5	73	6.9 [3.0-15.1]
	Cucu	2	82	2.4 [0.7-8.5]
	Wiloo	3	59	5.1 [1.7-13.9]
	Raa	3	86	3.5 [1.2-9.8]
	Nguu	3	95	3.2 [1.1-8.9]
**Total**		**64**	**1,389**	**4.6 [3.6-5.8]**
