# Supplementary material for: High prevalence of epilepsy in onchocerciasis endemic health areas in Democratic Republic of the Congo
Source: Infect Dis Poverty. 2018 Aug 1;7:68. doi: 10.1186/s40249-018-0452-1 (PMC6069757; doi:10.1186/s40249-018-0452-1)
Supplement: Supplementary file 3 — Percentage of individuals with a positive OV16 antibody test result by village. (PDF 11 kb) [file 40249_2018_452_MOESM3_ESM.pdf]

Additional file 2. Percentage of individuals with a positive OV16 antibody test result by village

Health area	Village	N of OV16+	N of OV16-	% of OV16+ [95% CI]
**Draju**	Nzuru	29	52	35.8 [26.2-46.7]
	Ruju	11	21	34.4 [20.4-51.7]
	Kpana	18	42	30.0 [19.9-42.5]
	Umulo	9	23	28.1 [15.6-45.4]
	Makala	12	16	42.9 [26.5-60.9]
	Ndroy	6	10	37.5 [18.5-61.4]
	Mbesi	17	8	68.0 [48.4-82.8]
	Yau	17	42	28.8 [18.8-41.4]
	Draju	9	9	50.0 [29.0-71.0]
	Kondu	9	11	45.0 [25.8-65.8]
	Nyodu	2	8	20.0 [5.7-51.0]
	Jupadrogo	16	36	30.8 [19.9-44.3]
**Kanga**	Kanga	20	67	23.0 [15.4-32.9]
	Juparima	19	60	24.1 [16.0-34.5]
	Jabi	18	28	39.1 [26.4-53.5]
	Djambu	15	38	28.3 [18.0-41.6]
	Cucu	16	31	34.0 [22.2-48.3]
	Wiloo	18	24	42.9 [29.1-57.8]
	Raa	11	44	20.0 [11.6-32.4]
	Nguu	6	64	8.6 [4.0-17.5]
**Total**		**278**	**634**	**30.5 [27.6-33.6]**
